# Metabolic reprogramming of immune cells: Shaping the tumor microenvironment in hepatocellular carcinoma

**DOI:** 10.1002/cam4.4177

**Published:** 2021-08-13

**Authors:** Yujia Xia, Zachary J. Brown, Hai Huang, Allan Tsung

**Affiliations:** ^1^ Division of Surgical Oncology Department of Surgery The Ohio State University Wexner Medical Center Columbus OH USA; ^2^ Department of Gastroenterology Tongji Hospital Tongji Medical College Huazhong University of Science and Technology Wuhan Hubei China

**Keywords:** hepatocellular carcinoma, immune cells, metabolic reprogramming, tumor immunology, tumor microenvironment

## Abstract

Hepatocellular carcinoma (HCC) is a typical inflammation‐induced cancer and displays a complex interaction between the tumor microenvironment and tumor development. Immune cells in the HCC microenvironment play both pro‐ and anti‐tumoral roles in HCC progression. An increasing number of findings indicate that metabolic reprogramming is essential for immune cell differentiation and function. In this review, we discuss the metabolic changes of different immune cells and correlate these findings to HCC progression.

## Graphical abstract

Immune cells in the Hepatocellular carcinoma (HCC) microenvironment play both pro‐and anti‐tumoral roles in HCC progression. An increasing number of findings indicate that metabolic reprogramming is essential for immune cell differentiation and function. In this review, we discuss the metabolic changes of different immune cells and correlate these findings to HCC progression.

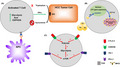

## 1 INTRODUCTION

Hepatocellular carcinoma (HCC) is by far the most common primary liver cancer and also the fifth leading cause of cancer‐related mortality worldwide.^1^, ^2^ Despite recent findings with targeted and immune therapies, the prognosis of patients at an advanced HCC stage remains poor with limited options for systemic therapy and high recurrence rates after locoregional therapies.^3^, ^4^, ^5^, ^6^ The tumor microenvironment (TME) consists of an intensive interplay between tumor cells, stroma, and immune cells with all these interactions being critical in tumorigenesis, metastasis, and drug resistance.^3^, ^7^ The initiation and progression of HCC are highly related to chronic inflammation resulting in a complex role of immune cells in cancer‐related inflammation and tumor cell immune evasion with HCC progression, angiogenesis, and eventual metastasis.^3^, ^8^, ^9^, ^10^, ^11^, ^12^ In addition, liver diseases can have varying effects on the TME some of which are not fully understood.^13^, ^14^, ^15^

**FIGURE 1:**
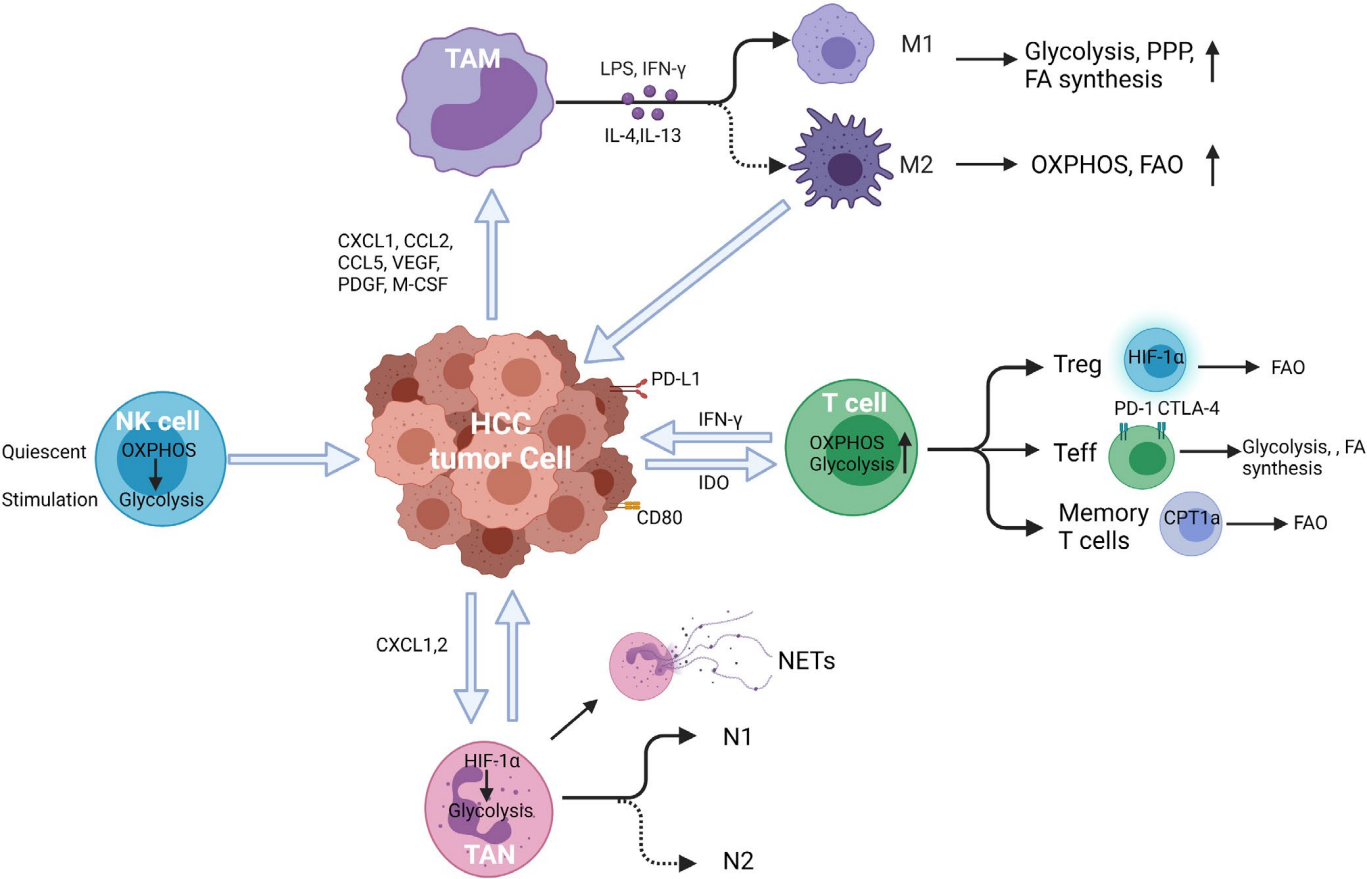
Metabolic reprogramming of immune cells in tumor microenvironment. Metabolic alterations in the indicated immune cells (macrophage, neutrophil, NK cells, and T cells) are presented

Cellular metabolism and basal metabolic needs are critical to cell growth, development, migration, and tissue‐specific functions.^16^ Metabolic reprogramming has been shown as a significant sign of cancer progression, not only affecting the survival and proliferation signals within tumor cells themselves but also altering the TME.^14^, ^17^, ^18^, ^19^, ^20^, ^21^ Accumulating evidence indicates that metabolic processes regulate the phenotype, function, and survival of immune cells.^14^, ^17^, ^21^, ^22^, ^23^, ^24^, ^25^ In this review, we discuss metabolic reprogramming in innate and adaptive immune cells (Figure 1), and their functional effects on HCC progression and metastasis. (Table 1).

**TABLE 1:** Metabolism and function of immune cells

Immune cell lineage	Subtypes	Function	Metabolism
Macrophage			
	M1 (classical activation)	Pathogen clearance and antigen presentation Secretes pro‐inflammatory cytokines and express high levels of MHC	Enhanced glycolysis, PPP, and FA synthesis
	M2 (alternate activation)	Produce anti‐inflammatory cytokines to promote immunosuppression and tumor progression	Increased OXPHOS and FAO decreased glycolysis and PPP
Neutrophil			Glycolysis If exposed to a TME deficient in glucose, adapt to mitochondrial FAO
	N1 (anti‐tumor)	Anti‐tumor polarization induced by type 1 IFNs	
	N2 (pro‐tumor)	TGF‐β an overexpressed by tumor cells polarize neutrophils to pro‐tumor phenotype	
	Neutrophil extracellular traps (NETs)	Fibers of decondensed DNA	Dependent on glucose and to a lesser extent on glutamine
NK cell		Critical in the early immune response regulating the adaptive immune response through the release of IFN‐γ	Use OXPHOS during their resting state and upon short‐term activation Prolonged stimulation: switch to glycolysis
T cell			
	CD4^+^ Helper T cells	Mediator of immune function secreting cytokines to heighten immune response	Glycolysis and ACC‐mediated de novo FA synthesis
	CD8^+^ Cytotoxic T cells	Direct cytotoxic killing of cancer cells	Enhanced glycolysis, glutaminolysis, and FAO to exert anti‐tumor cytotoxicity
	Regulatory T cells	Dampen the immune response	FAO rather than glycolysis
	Memory T cells	Protection against reinfection or tumor re‐emergence	Mitochondrial FAO for development and long‐term survival

## 2 INNATE IMMUNE SYSTEM

### 2.1 Tumor‐associated macrophages

Tumor‐associated macrophages (TAMs) are a prominent component of the TME and are often considered a biomarker of poor clinical prognosis.^26^, ^27^ TAMs have long been considered to be recruited to the tumor by chemokines such as CXCL1, CCL2, CCL5, and growth factors (VEGF, PDGF, and M‐CSF).^28^, ^29^, ^30^ TAMs act a critical role in innate and adaptive immunity by recruiting other immune cell types and act as antigen‐presenting cells. However, tumors may be able to alter the normal developmental process of TAMs to express PD‐L1 in the TME of HCC where increased expression of PD‐L1 was linked to disease progression and mortality.^27^

TAMs have a high functional plasticity with two activation phenotypes: classical (M1) and alternative (M2). M1 polarization can be stimulated by IFN‐γ alone or together with bacterial moieties, such as the lipopolysaccharide (LPS). The M1 phenotype produces several pro‐inflammatory cytokines and effector molecules, such as TNF‐α, IL‐23, IL‐12, and IL‐6, major histocompatibility complex (MHC)‐related molecules, such as class I and II, and iNOS. M1 and M2 subtypes exert contrasting effects in inflammation and tumor development. M1 cells function in pathogen resistance and tumor control. However, M2 macrophages release a great number of immunosuppressive cytokines such as arginase 1 (ARG1), TGF‐β, and IL‐10 to promote immunosuppression, tumor progression, and contribute to resistance to chemotherapies.^20^, ^30^ Therefore, the balance between M1 and M2 in TAMs is important for cancer immune therapy.

TAMs are frequently found in the stroma of HCC and are polarized to the M2‐like phenotype.^31^ M2‐like TAMs in HCC produce immunosuppressive functions with an enhancement of cancer proliferation, invasion, metastasis, extracellular matrix (ECM) remodeling, angiogenesis, epithelial–mesenchymal transition (EMT), and cancer cell stemness maintenance.^32^, ^33^, ^34^ In addition, M2 macrophages have been linked to early tumor recurrence. Krall et al showed that the process of postoperative wound healing leads to myeloid cells mobilization and the shift of TAMs to M2 phenotype, contributing to the immunosuppressive TME and mediating surgery‐induced tumor outgrowth.^35^

**FIGURE 2:**
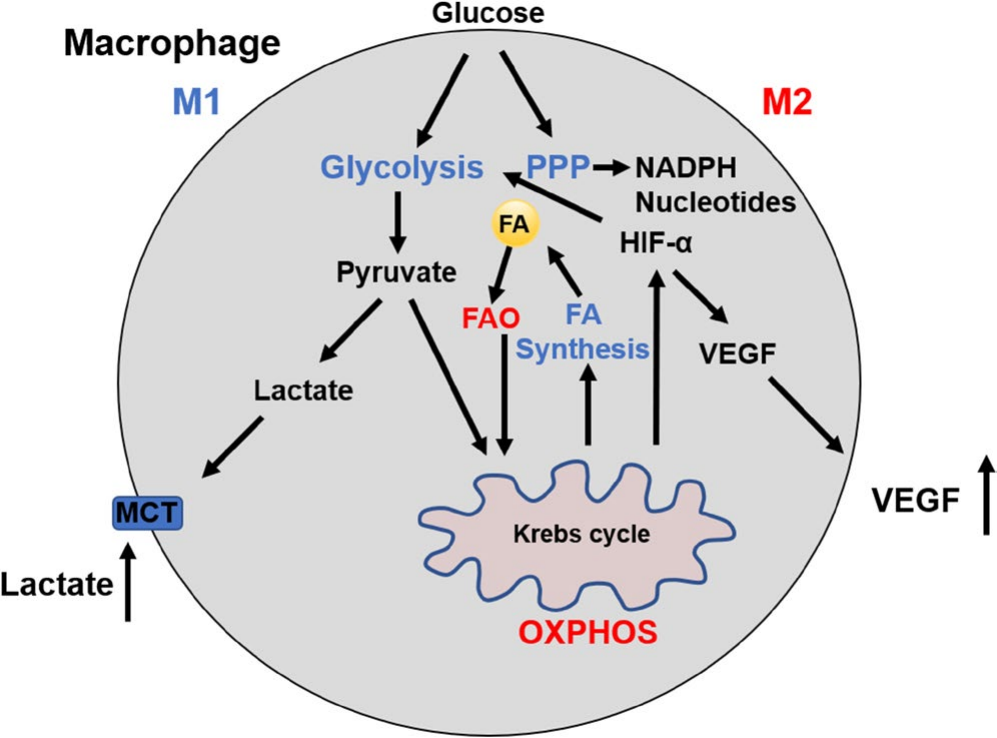
Metabolism in M1 and M2 macrophages. M1 macrophages display an enhanced glycolysis, PPP, FA synthesis, a decreased OXPHOS, and TCA cycle. Activation of glycolysis which results in increased production of lactate, increased FA uptake and synthesis, and increased uptake of glutamine to fuel the TCA cycle. M2 cells are well known as anti‐inflammatory macrophages with enhanced OXPHOS and activated FAO, reduced glycolysis, and PPP

Altered cellular metabolism appears to be responsible for the polarization and function in activated macrophages.^36^ M1 macrophages display pro‐inflammatory properties defined by an enhanced glycolysis, metabolic flux through pentose phosphate pathway (PPP), fatty acid (FA) synthesis, a decreased oxidative phosphorylation (OXPHOS), and tricarboxylic acid (TCA) cycle. M2 cells are well known as anti‐inflammatory macrophages with enhanced OXPHOS and activated fatty acid oxidation (FAO), reduced glycolysis, and PPP (Figure 2). ^37^, ^38^, ^39^ These metabolic changes in TAMs not only regulate energy production but also mediate the variety of TAM phenotypes. M1 macrophages fail to transform into an M2 phenotype after the stimulation of IL‐4 in vitro and in vivo secondary to M1‐associated inhibition of mitochondrial OXPHOS.^40^ Metabolic changes promote the development of tumor where M2 macrophages lead to increased proliferation, invasion, and metastasis of HCC cells via FAO pathway by enhancing IL‐1β secretion.^41^

TAMs also have been found to have altered glucose metabolism, and glucose metabolism varies at different stages of carcinogenesis. At the early inflammatory phase of cancer onset, the main energy source of TAMs is through glycolysis; whereas at the later stage of tumor progression TAMs utilize OXPHOS.^42^, ^43^ This shift in energy sources is mediated by lactic acid and cytokines. Tumor‐derived lactate also contributes to the polarization of TAMs, which is mediated through hypoxia‐inducible factor‐1α (HIF‐1α). Lactate induces ARG1 expression in TAMs and consequently affects the tumor cell proliferation and collagen synthesis through the production of ornithine and polyamines.^44^ Lactic acid also induced a malignancy‐promoting phenotype by promoting vascular endothelial growth factor A (VEGFA) expression via triggering HIF‐1α activation.^45^ In addition, glycolysis inhibition in TAMs was able to reverse TAM‐induced angiogenesis, extravasation, and EMT.^46^ Furthermore, the increase in glycolysis in the TME secondary to monocytes and macrophages has been linked to PD‐L1 expression through the upregulation of the glycolytic enzyme PFKFB3 in TAMs.^47^ High levels of PD‐L1 on tumor cells and tumor‐infiltrating immune cells are a negative prognostic factor in tumors and has been found to be a predictor of recurrence.^48^, ^49^ Therefore, PFKFB3 can be a potential therapeutic target in both tumor cells and TAMs in HCC.^47^

### 2.2 Tumor‐associated neutrophils

Neutrophils has been recognized as the most abundant circulating immune cell type and the first‐line responders to many pathologic processes. However, while providing protection in some scenarios they can cause damage in others.^50^ It is known that tumors secrete chemokines that support the recruitment of leukocytes including neutrophils from the circulation into the TME thus producing tumor‐infiltrating neutrophils (TANs).^51^ TANs play a key role in promoting growth, invasion, angiogenesis, and metastasis in HCC.^52^ Neutrophils are predominantly found in the peritumoral stroma and the levels of TANs can serve as an independent predictor of poor outcome in HCC patients.^53^

Neutrophils derive most of their energy from glycolysis, which is oxygen‐independent energy generation allows neutrophils to function effectively at low oxygen levels.^54^, ^55^ HIF‐1α also known as a crucial regulator of metabolism in myeloid cells.^56^ HIF‐1α is essential for neutrophil glucose uptake after activation mediated by glucose transporter GLUT1 translocation to the cell surface. Enhanced glucose consumption has been shown to be crucial for enhanced neutrophil survival and function in a hypoxic microenvironment.^57^, ^58^ The TCA cycle, OXPHOS, FAO, and PPP play different roles in the energetic, biosynthetic, and functional requirements of neutrophils (Table 2).^59^, ^60^, ^61^, ^62^, ^63^, ^64^ Due to limited glucose, neutrophils utilize mitochondrial FAO to fuel NOX‐2‐dependent reactive oxygen species (ROS) production.^65^ Tumor‐elicited oxidative neutrophils are an important source of NADPH oxidase‐dependent ROS and are able to maintain immune tolerance in the nutrient‐limited TME. In addition, neutrophils from patients with cancer show increased immaturity, mitochondrial content, and OXPHOS.^65^

**TABLE 2:** Key metabolic pathways involved in different neutrophil functions

Neutrophil Function	Metabolic pathways
Differentiation	OXPHOS, FAO, and TCA
Phagocytosis	Glycolysis
ROS production	PPP and Glutaminolysis
Degranulation	Glycolysis
NETs	Glycolysis and PPP
Chemotaxis	Glycolysis and mitochondria

The thought that neutrophils are primarily dependent on the glycolytic pathway is currently being challenged as emerging research is indicating metabolic reprogramming may occur by mediators of inflammation, sepsis, diabetes, and cancer.^66^ In early stages of cancer, neutrophils display enhances migration with high rates of glycolysis and OXPHOS to produce more ATP.^67^ However, if exposed to a TME deficient in glucose, neutrophils adapt to mitochondrial FAO.^67^ Similar to macrophages, TANs can be classified into anti‐tumorigenic phenotype (N1) and pro‐tumorigenic phenotype (N2).^68^ However, the effect of metabolic reprogramming on the polarization of TANs is still largely unknown.

Recent studies have discovered a new function of neutrophils in cancer progression through the production of neutrophil extracellular traps (NETs). NETs are composed of sticky DNA fibers that are decorated with several proteins like myeloperoxidase and neutrophil elastase released from activated neutrophils.^69^ NET formation clearly plays an important role in HCC development associated with nonalcoholic steatohepatitis (NASH).^70^ NETs deletion did not affect liver steatosis and FFA accumulation but ameliorated macrophage infiltration and changed the inflammatory environment to one that is less favorable for the biological behavior of the HCC.^70^ Metabolically, NET formation is critically dependent on glucose and to a lesser extent on glutamine.^61^, ^62^ Future studies will be required to further investigate metabolic reprogramming in neutrophil polarization and its influence on the TME and NETs in tumor formation and progression.

### 2.3 Natural Killer (NK) cells

NK cells are key regulators of early immune reactions. They also control adaptive immunity through the secretion of cytokines, such as IFN‐γ, that impact dendritic cells, macrophages, and neutrophils.^71^, ^72^, ^73^ NK cells exhibit a superior capacity for expansion to create innate memory in parallel with the adaptive immune system. The number of NK cells was significantly lower in the liver with tumor, when compared with the adjacent normal liver of HCC patients.^74^, ^75^ Moreover, the absolute number of NK cells in peripheral blood and liver tumor tissues is positively correlated with the apoptosis rate and patient survival in HCC.^76^, ^77^, ^78^

The interaction of NK cells and tumor cells can induce the expression of CD25, which is identical to the α chain of the high‐affinity interleukin (IL)‐2 receptor. CD25^high^ NK cells are highly sensitive to IL‐2, leading to a robust and prolonged NK cell anti‐tumor response.^79^ As a result, NK cells show an increase in metabolic signaling pathways, nutrition transport cell growth as well as increased function and survival.^79^, ^80^, ^81^ NK cells require dramatic changes in metabolism upon activation. NK cells utilize OXPHOS during their metabolically quiescent state and after short‐term activation.^82^ However, upon prolonged stimulation NK cells switch to glycolytic metabolism.^83^ The mechanistic target of rapamycin (mTOR) exerts a crucial role in attaining a heightened glycolytic state. The mTOR genetic deletion inhibits NK cell effector function, inhibiting IFN‐γ production and granzyme B, which further strengthens the evidence linking cellular metabolism with NK cell function.^83^, ^84^, ^85^ It has been shown that energy metabolism and cell motility defects play pivotal roles in circulating NK cells, which impairs functional NK cell in HCC patients.^86^ However, the mechanisms that tumor‐derived stimuli regulate NK activation and energy metabolism in anti‐tumor immunity have yet to be established.

## 3 ADAPTIVE IMMUNE SYSTEM

**FIGURE 3:**
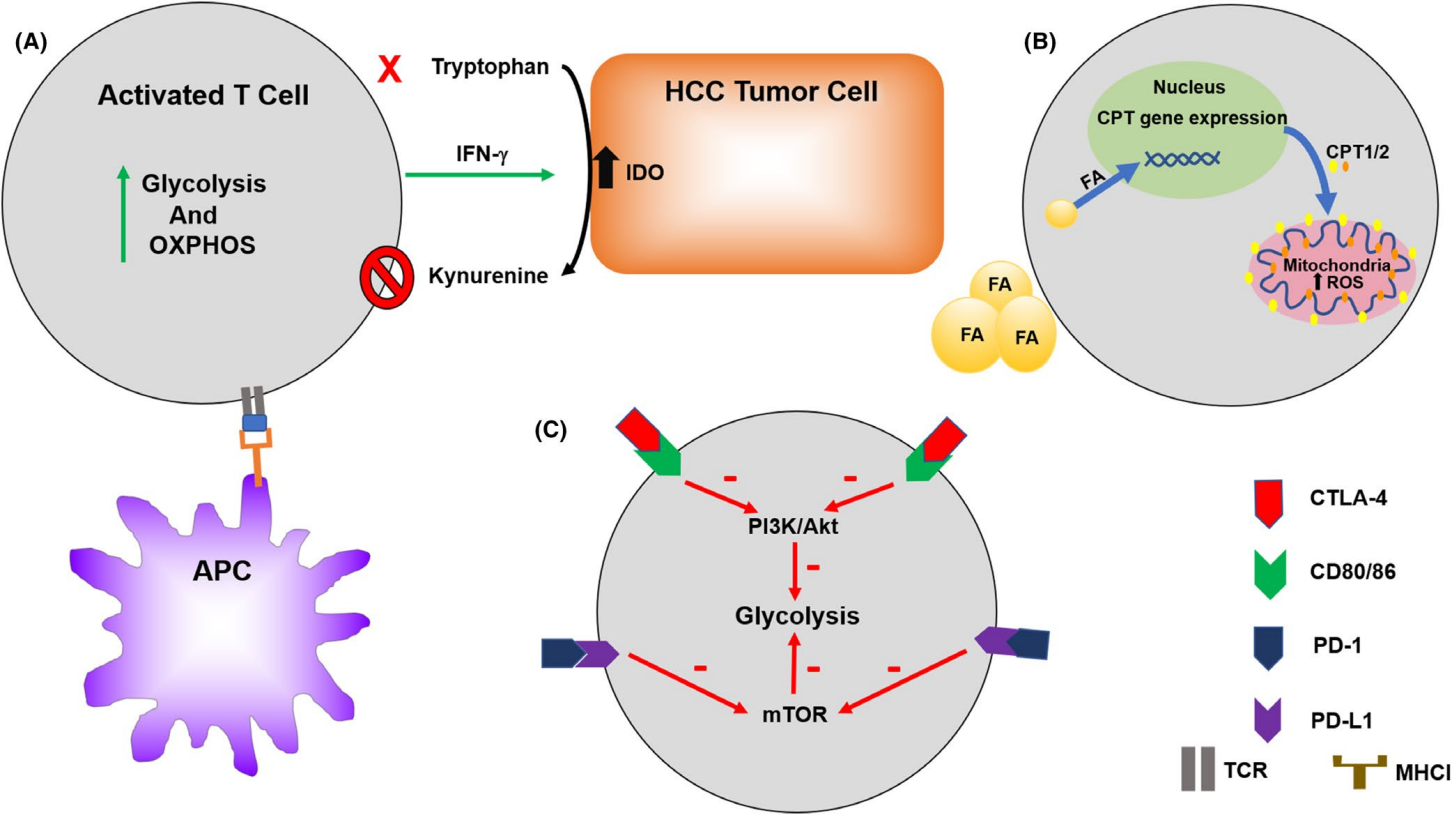
Regulation metabolism of T cells. (A) Activated T cells have increased metabolic processes to secrete activating cytokines such as IFN‐γ. However, tumor cells can have upregulation of alternate immune checkpoints to produce immune escape. (B) Fatty acids can increase the CPT gene expression causing increased ROS and CD4+ T‐cell death produce an immunosuppressive TME. (C) CTLA‐4 and PD‐1 immune checkpoints can alter T‐cell metabolism causing T‐cell anergy, exhaustion, and autophagy

The effect of T cells in the immunity to cancer, including HCC, is increasingly recognized.^87^ As immunotherapy is becoming more widespread for malignancies including HCC it is important to further investigate the role of T‐cell metabolism.^88^ T‐cell metabolic reprogramming processes are closely related to the differentiation and function of T cell (Figure 3). After antigen stimulation, T cells grow, proliferate, and activation occurs releasing cytotoxic factors and cytokines. Therefore, T cells undergo profound metabolic shifts to sustain the biosynthesis of nucleic acids, amino acids, and proteins. In order to meet the requirement for cell proliferation, T cells simultaneously increase glucose and glutamine metabolism.^89^, ^90^ Interestingly, T cells, like cancer cells, primarily utilize glycolysis even when oxygen is sufficient for OXPHOS.^91^, ^92^, ^93^, ^94^, ^95^ It is hypothesized this is due to provide other advantages to the cell other than mere energy production, such as to promote the acquisition of nutrients to produce daughter cells.^91^, ^96^, ^97^

Furthermore, distinct T‐cell subsets possess special metabolic alterations that are associated with their function and may provide a novel therapeutic strategy for immune regulation. Naïve T‐cell activation relies on OXPHOS while IFN‐γ cytokine production depends on glycolysis.^91^ Glycolysis and acetyl‐CoA carboxylase (ACC)‐mediated de novo fatty acid synthesis are the primary mechanisms to effector CD4^+^ T‐cell fate.^98^ GLUT1 knockdown reduces glucose uptake, transportation, and glycolysis, and also impairs the growth, proliferation, and prevented T‐effector (Teff) expansion.^99^ Cytotoxic CD8^+^ T cells increase glycolysis, glutaminolysis, and FAO to play effective anti‐tumor cytotoxic activity. mTOR is a central integration site for metabolic switch of CD8^+^ T cells.^100^, ^101^ Upon activation, PI3K/Akt/mTOR, and the transcription factor c‐Myc‐related pathways are activated in CD8^+^ T cells, as well as Th1, Th2, and Th17 cells, to regulate glycolytic genes such as GLUT1, PDK1, or HK2.^89^, ^90^, ^102^, ^103^

Regulatory T cells (Tregs), which depend mainly on FAO for energy and minimally utilize glycolysis, can survive under nutrient‐depleted tumor environments and play an immunosuppressive effect.^99^, ^104^ The inhibition of T‐cell intrinsic mTORC1 by rapamycin dampens growth and proliferation of Teff cell and reinforces Treg generation.^105^, ^106^, ^107^ HIF‐1α has been suggested to be the main regulator in T‐cell fates, resulting in increased Treg development and suppressed Th17 cells.^108^, ^109^, ^110^ How HIF‐1α mediates Th17 and Treg cell differentiation is unclear, but it has been shown to be involved in both metabolic and nonmetabolic processes. Metabolically, blocking glycolysis by HIF‐1α disturbs the Th17/Treg balance. Nonmetabolically, HIF‐1α activates the transcription factor RORγt and collaborates with RORγt to promote Th17 development. Interestingly, HIF‐1α can directly bind Foxp3 protein and impact its degradation.^110^ The metabolic switch of hypoxia and HIF‐1α play important roles in T cells and will be crucial for more studies in the future.

Another fundamental ability of the adaptive immune system is to generate a pool of long‐lived, antigen‐specific memory T and B cells. These memory cells protect against reinfection or tumor recurrence. Unlike activated effector T cells, memory T cells have dramatic changes in metabolism which does not require high rates of anabolism to maintain rapid proliferation. However, they still need energy generation to conduct certain basic cellular functions and stay alive. The metabolic switch from aerobic glycolysis to mitochondrial FAO is important for development and long‐term survival.^111^, ^112^ In contrast to effector T cells, memory T cells have a quite distinct metabolic state, which reduces glycolysis, maintains greater mitochondrial metabolism, and use fatty acids.^111^ Memory T cells induce expression of the critical mitochondrial transporter CPT1a, and this protein increases the memory T cells generation and survival.^111^

As immunotherapy is becoming more widely used in HCC, it is imperative to understand the relationship between tumor immunology and metabolism.^113^ On CD8^+^ and CD4^+^ T cells, isolated from HCC tissues, there is an up‐regulation of the immune checkpoint inhibitory proteins PD‐1, CTLA4, TIM3, and LAG3, compared with T cells from tumor‐free liver tissues.^114^ Several studies have demonstrated that PD‐1 and CTLA‐4 engagements inhibit glucose metabolism, thereby impairing T‐cell activation and ultimately inducing T‐cell dysfunction (anergy, tolerance, and exhaustion). CTLA‐4 has been shown to block Akt phosphorylation, thereby reducing glycolysis that is supported for cell growth and function.^93^, ^115^, ^116^ Meanwhile, ligand binding to PD‐1 can antagonize mTOR signaling via Akt and PI3K inhibition, thus diminishing glycolysis and glutaminolysis within the target cells.^117^ Taken together, increasing evidence suggests that metabolic reprogramming exert a key effect on anti‐ and pro‐tumor immunity of tumor‐infiltrating lymphocytes.^118^

The tumor microenvironment is not only the crucial regulator in HCC development but perhaps also a response to therapy. Nonalcoholic fatty liver disease (NAFLD) and its advanced form nonalcoholic steatohepatitis (NASH) are an increasing problem worldwide as the prevalence of obesity increases. The accumulation of fatty acids stored in liver tissue has been shown to cause selective CD4^+^ helper T cells loss.^13^ This is believed to be due to upregulate CPT family genes that induced by fatty acid in the more mitochondria‐rich CD4^+^ T cells and increase in reactive oxygen species.^13^, ^14^ A T cell‐dependent immunotherapy was found to be no longer effective in mice with liver tumors in the setting of fatty liver disease.^119^ Additionally, tryptophan is an essential amino acid and its deprivation has been shown to cause T cells derrangements.^120^ Indoleamine 2,3‐dioxygenase (IDO) is an intracellular enzyme that selective cleavage tryptophan and is overexpressed in the TME. IDO can render T cells without the essential amino acid as well as producing an immunosuppressive phenotype with an increase in Tregs.^121^ IDO production in HCC has been linked to a poor prognosis.^121^ In mouse models, IDO has been shown to be induced in HCC tumors providing immune escape from immune checkpoint inhibitors. This phenomenon could be substantially reversed by the administration of an IDO inhibitor along with the immune checkpoint inhibitor.^21^

## 4 CONCLUSION

In recent years, many advances have been obtained in understanding the effects and mechanisms of tumor‐infiltrating immune cells in the development of HCC. Metabolic reprogramming affects the differentiation stages and functions of tumor‐associated immune cells in the TME and thus plays an important role in the progression of HCC. Immune cells use diverse metabolic pathways including glycolysis, FAO, TCA cycle, PPP, and glutaminolysis to perform distinct functions. The lack of nutrients from the environment suppresses antitumor immune cells, such as M1 macrophages, N1 neutrophils, and CD8^+^ T cells, promoting the differentiation and activation of pro‐tumor immune cells, including M2 macrophages, N2 neutrophils, and Tregs. All of these features make the immune metabolism in TME a powerful target for cancer therapy. However, numerous findings indicate that the roles of immune cells under different conditions are self‐contradictory, for which many of the mechanisms are still unknown. Future studies are required to unravel the complex interplay between metabolic reprogramming, the immune system, and the TME and increased understanding will prove beneficial in advancing therapeutic options for patients with HCC.

## CONFLICT OF INTEREST

The authors made no disclosures.

## ETHICAL STATEMENT

This study does not involve any human or animal testing.

## Data Availability

This is a review article and therefore the data discussed in this study are publicly available. No ethical approval was required.
